# The effect of pharmacological inhibition of Serine Proteases on neuronal networks *in vitro*

**DOI:** 10.7717/peerj.6796

**Published:** 2019-04-23

**Authors:** Sebastiaan Van De Vijver, Stephan Missault, Jeroen Van Soom, Pieter Van Der Veken, Koen Augustyns, Jurgen Joossens, Stefanie Dedeurwaerdere, Michele Giugliano

**Affiliations:** 1 Molecular, Cellular, and Network Excitability, Department of Biomedical Sciences and Institute Born-Bunge, University of Antwerp, Wilrijk, Flanders, Belgium; 2 Experimental Laboratory of Translational Neuroscience and Otolaryngology, Department of Translational Neurosciences, University of Antwerp, Wilrijk, Flanders, Belgium; 3 Laboratory of Medicinal Chemistry, Department of Pharmaceutical Sciences, University of Antwerp, Wilrijk, Flanders, Belgium; 4 Neuroscience sector, Scuola Internazionale Superiore di Studi Avanzati, Trieste, Italy; 5 Laboratory of Experimental Haematology, VAXINFECTIO, University of Antwerp, Wilrijk, Flanders, Belgium

**Keywords:** Extracellular matrix, Microelectrode arrays, Neurons, Network activity, Serine proteases, Patch-clamp, Cortex, Electrophysiology, Synaptic connectivity, Neurobiology

## Abstract

Neurons are embedded in an extracellular matrix (ECM), which functions both as a scaffold and as a regulator of neuronal function. The ECM is in turn dynamically altered through the action of serine proteases, which break down its constituents. This pathway has been implicated in the regulation of synaptic plasticity and of neuronal intrinsic excitability. In this study, we determined the short-term effects of interfering with proteolytic processes in the ECM, with a newly developed serine protease inhibitor. We monitored the spontaneous electrophysiological activity of *in vitro* primary rat cortical cultures, using microelectrode arrays. While pharmacological inhibition at a low dosage had no significant effect, at elevated concentrations it altered significantly network synchronization and functional connectivity but left unaltered single-cell electrical properties. These results suggest that serine protease inhibition affects synaptic properties, likely through its actions on the ECM.

## Introduction

The extracellular matrix (ECM) provides physical support and a stable environment to neurons *in vivo*. As a physical barrier, ECM may limit or refine structural connectivity between cells, while at the same time also functioning as a critical regulator of synaptic plasticity (for a review, see Mukhina, Korotchenko & Dityatev, 2012; Dityatev & Schachner, 2003; Dityatev, Schachner & Sonderegger, 2010). The enzymatic removal of the ECM in *in vitro* cultures has been reported to facilitate the rearrangement of the neuronal connectivity profile (Bikbaev, Frischknecht & Heine, 2015). Proteases are known to play an essential role in this regulation (reviewed in Ferrer-Ferrer & Dityatev, 2018). Through their proteolytic action, they mould the structure of the ECM, which allows morphological changes to occur. Moreover, the extracellular proteolysis releases signalling molecules from the ECM, such as trans-synaptic proteins and growth factors, which are known to affect synaptic plasticity (Tsilibary et al., 2014). This evidence is especially strong for several serine proteases (SP) and matrix metalloproteinases, as reviewed in Tsilibary et al. (2014) and Shiosaka (2004).

An important regulator of extracellular metabolism is the plasminogen-plasmin system. It is thought to be involved in structural remodelling and could thus affect neuronal connectivity. Among SP, the urokinase-type plasminogen activator (uPA) converts plasminogen to plasmin, which in turn is responsible for the degradation of several extracellular proteins, both directly and indirectly (for a review, see  Smith & Marshall, 2010). However, relatively little is known about the links between uPA and synaptic connectivity under physiological conditions in neuronal microcircuits. uPA has for instance been implicated in plasticity occurring during peripheral nerve regeneration (Seeds et al., 2011; Karagyaur et al., 2015; Minor & Seeds, 2008), as well as in dendritic spine recovery following ischemic stroke (Wu et al., 2014). uPA overexpression in transgenic animals has been associated with negative effects on learning and memory (Meiri et al., 1994). In addition to its links to plasticity, a polymorphism in the uPA gene is correlated with higher A*β* plaque counts in Alzheimer patients (Riemenschneider et al., 2006). Thus, any investigation of the SP involvement in neuronal networks electrophysiology might be beneficial for clarifying the physiological aspects related to the involvement of this gene.

Neuropsin, also known as kallikrein-related peptidase 8 (KLK8), is another important SP that is expressed in an activity-dependent manner and secreted in the extracellular space as an inactive zymogen. Neuropsin is converted to its active form in an NMDA-receptor-activity-dependent manner (Matsumoto-Miyai et al., 2003). Through cleavage of its target transmembrane and extracellular proteins (i.e., fibronectin, NCAM-L1, EphB2 and neuregulin-1 (Alexander, Bernhard & Asla, 2014)), it activates a signalling pathway involving AMPA receptors, ErbB4, vitronectin, integrin and L-type voltage-dependent calcium ion channels (Matsumoto-Miyai et al., 2003; Ishikawa et al., 2008; Tamura et al., 2012; Kawata et al., 2017; Tamura et al., 2006). Many of these targets are known for their clear involvement in structural and functional synaptic plasticity, and neuropsin has been implicated by several lines of evidence in the regulation of synaptic plasticity (Mukhina, Korotchenko & Dityatev, 2012). Current data demonstrate that it modulates the early phase of long-term potentiation (LTP) (Tamura et al., 2006; Komai et al., 2000), occurring independently of protein synthesis, that it enhances neurite outgrowth and fasciculation during development (Oka et al., 2002), that it primes synapses through tagging, making them more susceptible to persistent LTP (Ishikawa et al., 2008; Ishikawa, Tamura & Shiosaka, 2011), and that it strengthens GABAergic transmission (Tamura et al., 2012). Recently, neuropsin has also been shown to affect the excitation-inhibition (E-I) balance in the hippocampus, through its modulation of parvalbumin-expressing interneuron activity via neuregulin-1 and ErbB4 signalling (Kawata et al., 2017). Moreover, an increased hyperexcitability to seizure-evoking stimuli (Davies et al., 2001) was reported in neuropsin-deficient mice, with decreased GABAergic interneuron activity and increased pyramidal neuron activity following administration of the convulsant kainic acid (Kawata et al., 2017).

**Figure 1:**
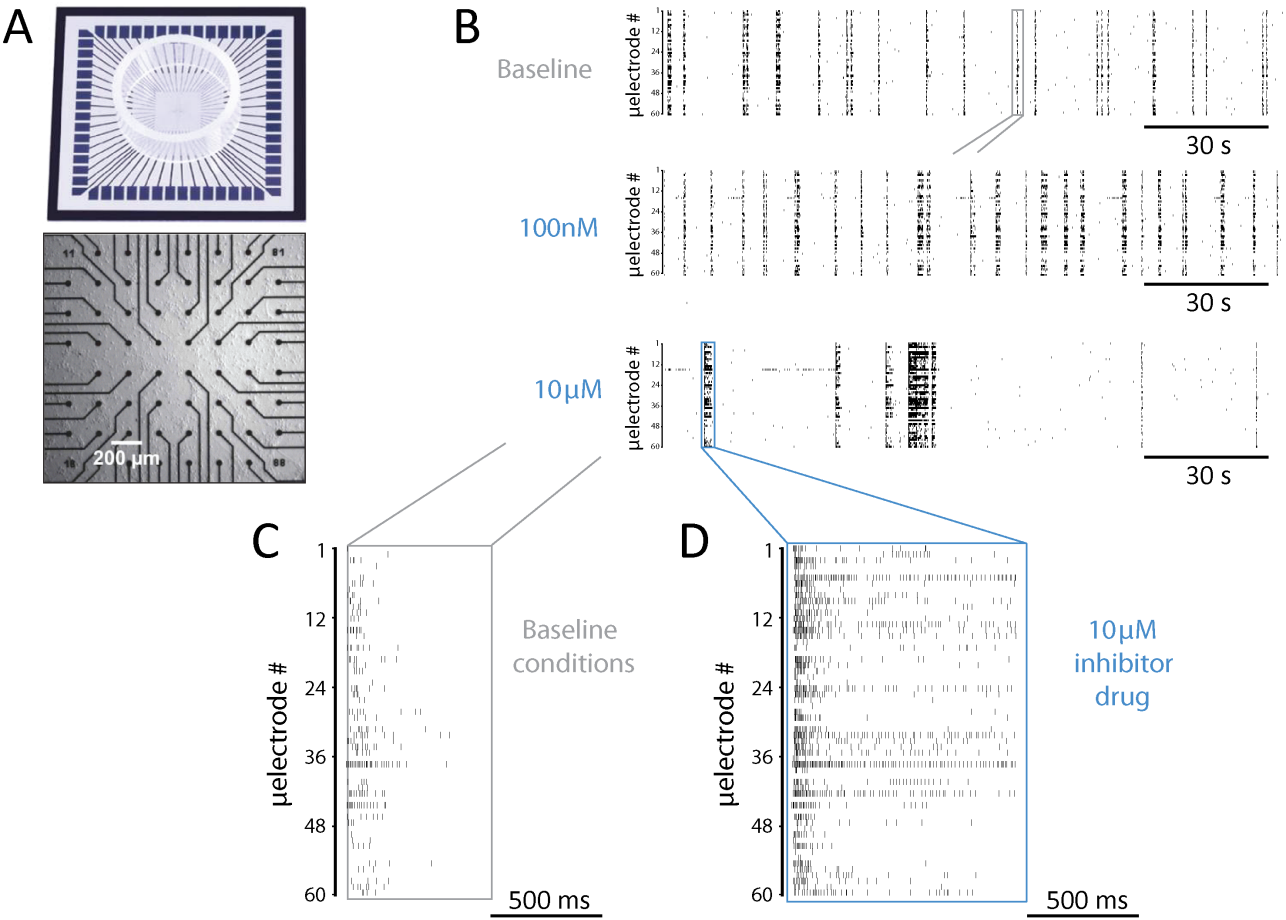
Experimental setup and a representative example of network-wide activity under SP inhibition. (A) *In vitro* rat cortical networks were grown on substrate-integrated arrays of microelectrodes (MEAs) and their spontaneous electrical activity was monitored from up to 60 spatial locations. (B–F) Data from a representative experiment are shown as *raster plots*: each row refers to a distinct microelectrode, and each small marker represents the time of occurrence of a spike detected at that microelectrode (B–D). Three sample raster plots are shown from the same MEA, documenting baseline activity (top), low dose (middle), and high dose (bottom) of the SP inhibitor. At high dose, the occurrence frequency of NBs decreased, their timing variability increased, and their duration (compare E and F) increased. (E–F) A magnification over the NBs in B highlighted in grey and blue, reveals clearly the observed increase in NB duration.

In this study, we investigated a newly developed SP inhibitor (i.e., UAMC-01162, patent WO2007045496, the HCl salt of UAMC-00150/compound 6c in Joossens et al., 2007). This compound inhibits several SP, among which it has the highest affinity for rat uPA and neuropsin (Davies et al., 2001). Given the downstream ECM targets of uPA and neuropsin discussed above, inhibition of the extracellular SP is potentially capable of inducing changes in the network connectivity through ECM modification. We based our work on the hypothesis that such an effect can be studied in an *in vitro* model system of cortical microcircuits, as both uPA and neuropsin are known to be expressed in the rat cortical tissue  (Missault et al., 2017). Specifically, we asked whether such an effect can be observed from changes in the pattern of spontaneous electrical activity, measured non-invasively and over extended periods of time, by substrate-integrated microelectrode arrays (MEAs; Fig. 1). Our SP inhibitor consists of a diphenyl phosphonate group, which binds covalently to the serine alcohol in the catalytic centre of serine proteases and results in a phosphorylated and inhibited enzyme, and a benzylguanidine group, which provides high potency towards uPA and neuropsin (Missault et al., 2017). By extracellularly monitoring the electrical activity of neuronal networks by MEAs (Figs. 1B–1F), underlying changes in connectivity can be assessed, while still preserving a functioning ECM (Bikbaev, Frischknecht & Heine, 2015; Dityatev et al., 2006; John et al., 2006). By also intracellularly probing single-cell excitable properties by patch-clamp, the specificity of action of our inhibitor on neuronal synaptic coupling can be further supported. Here, we present our experimental findings, related to the episodic and spontaneous synchronisation of “bursts” of action potentials (Figs. 1E–1F) fired by neurons simultaneously across the network (i.e., hereafter referred to as network bursts). We demonstrate how the SP inhibition results in altered network electrophysiology and effective connectivity, while leaving single-cell properties unaffected.

## Materials and Methods

### Neuronal cultures

Primary cultures of mammalian neurons, dissociated from the postnatal rat neocortex, were prepared as described previously (Reinartz et al., 2014), in accordance with international and institutional guidelines on animal welfare. All procedures were approved by the Ethical Committee of the University of Antwerp (permission no. 2011_87)”- and licensed by the Belgian Animal, Plant and Food Directorate-General of the Federal Department of Public Health, Safety of the Food Chain and the Environment (license no. LA1100469). Recordings have been pooled over MEAs plated during several culturing sessions. Each culturing session was carried out using P0 rats from the same nest, although the gender of the P0 rats was left undetermined. The experiments originated from the use of 32 pups over 4 culturing sessions. The patch-clamp recordings were performed on 23 coverslips, originating from 12 pups over 3 culturing sessions. MEAs with a regular 8 × 8 arrangement of 60 titanium nitrate (TiN) microelectrodes, each with 30 µm diameter and 200 µm spacing (60MEA200/30iR-ITO-gr, MultiChannel Systems, Reutlingen, Germany), were employed in this study (Fig. 1A) in addition to conventional glass coverslips. Prior to cell seeding, each MEA or coverslip was coated overnight with polyethyleneimine (0.1% wt/vol in milli-Q water at room temperature), and then extensively washed with milli-Q water and air-dried. On MEAs, 1.8 × 10^6^ cells were seeded in a volume of 1 mL while, on coverslips, 3.5 × 10^5^ cells were seeded in 360 µL. After giving cells sufficient time to settle on the glass coverslip, medium was added to reach a final volume of 1 mL. This resulted in a plating density of 6’500 cells/mm^2^ and 2’000 cells/mm^2^ for MEAs and coverslips, respectively. After seeding, MEAs and coverslips were maintained in a conventional incubator at 37 °C, 5% CO_2_, and 100% relative humidity (R.H.) (5215, Shellab, Cornelius, OR, USA). MEAs were sealed with fluorinated Teflon membranes (Ala-MEA-Mem, Ala Science, Farmingdale, NY, USA) during the entire duration of the experiments, to reduce the risk of contamination, prevent water evaporation, and ensure gas exchanges. From DIV1 onwards, half of the culture medium volume was replaced every 2 days with fresh, pre-warmed medium. The medium consisted of MEM, 5% heat-inactivated horse serum, 20 mM glucose, 50 µg/mL gentamicin and 100 µM L-glutamine. From DIV7 onwards, the L-glutamine concentration in replacement medium was halved. All reagents were obtained from Sigma-Aldrich (St. Louis, MO, USA) or Life Technologies (Ghent, Belgium). We also repeated some of the experiments under serum-free conditions, as detailed in File S1.

### Pharmacology and experimental protocols

For MEA experiments, the SP inhibitor UAMC-01162—originally discussed in Joossens et al. (2007) as a TFA salt under the name UAMC-00150 (compound 6c), and in the present study complexed with HCl—was dissolved in 2% DMSO and added to the cultures in two incremental “concentration steps”. One hour of baseline spontaneous activity was first recorded, prior to applying the chemical compound. Subsequently, a small fraction of the culture medium was replaced with the SP inhibitor solution resulting in a final concentration of 100 nM, and the spontaneous electrical activity was recorded for one hour. Lastly, the final concentration of the compound was raised to 10 µM and the spontaneous electrical activity was recorded for one additional hour. At each step of compound addition to the bath, cultures were allowed to equilibrate for 10 min before starting the recordings. Control cultures were treated in the same manner, employing a DMSO solution without the inhibitor. The final DMSO concentration in the culture was 0.001% and 0.1% for the two concentration steps, respectively. For coverslips, the inhibitor was dissolved in 2% DMSO and added to the extracellular solution (i.e., described below) at a concentration of 10 µM. During patch-clamp intracellular experiments, the solution was continuously perfused at the rate of 1 mL/min and, for the control cultures, it contained DMSO without the inhibitor. Patch-clamp recordings started at least 30 min after initiating the perfusion of the inhibitor.

Finally, in some experiments, Gabazine (GBZ, SR-95531) was bath-applied prior to the start of MEA extracellular recordings at the final concentration of 30 µM, as a selective competitive blocker of GABA_A_ receptors. This drug was obtained from Abcam (Cambridge, England, UK).

### Extracellular electrophysiological recordings

At full maturity of the cultures, between DIV27 and 30, extracellular electrical recordings were made by MEAs using a commercial amplifier (MEA-1060-Up-BC, MultiChannel Systems GmbH, Reutlingen, Germany) with a 1–3’000 Hz bandwidth and an amplification factor of 1’200. The raw voltage waveforms, acquired from each of the 60 microelectrodes, were sampled at 25 kHz/channel and digitised at 16 bits by an A/D electronic board (MCCard, MultiChannel Systems), and stored on disk by the MCRack software (Multi Channel Systems) for subsequent analysis.

### Intracellular electrophysiological recordings

Glass coverslips were employed for patch-clamp experiments in cultures of 13 to 20 DIV, performed using an Axon Multiclamp 700B amplifier (Molecular Devices, USA), controlled by the LCG software (Linaro, Couto & Giugliano, 2014). Patch pipettes were pulled on a horizontal puller (P97, Sutter, Novato, USA) from filamented borosilicate glass capillaries (World Precision Instrument Inc. (WPI), USA), and had a resistance of 3.7–6.4 MΩ, when filled with an intracellular solution containing (in mM): 125 K-gluconate, 5 KCl, 4 NaCl, 1 CaCl_2_, 2 MgCl_2_, 10 EGTA, 10 HEPES, 4 Mg-ATP, 0.3 Na_2_-GTP, 10 Na_2_-phosphocreatine (pH adjusted to 7.4 with NaOH). An extracellular solution, containing (in mM): 145 NaCl, 4 KCl, 1 MgCl_2_, 2 CaCl_2_, 5 HEPES, 2 Na-pyruvate, 5 glucose (pH adjusted to 7.4 with NaOH), was constantly perfused at a rate of 1 mL/min and at a temperature of 37 °C for each experiment. In all the experiments, offline digital compensation of the electrode artifacts was obtained using the Active Electrode Compensation technique (Brette et al., 2008), implemented by LCG.

### Data analysis and statistics

All data processing was performed using custom-written MATLAB scripts (The MathWorks, Natik, MA, USA). Action potential and network-burst detection was carried out using the QSpike Tools package (Mahmud et al., 2014). In short, spike detection by peak detection was preceded by a band-pass filtering of the raw traces, and the threshold was adapted on the basis of the median of the raw voltage amplitude. All subsequent analyses were performed on the timing of each threshold-crossing event, which represented the occurrence of a putative action potential detected at any given microelectrode of the array (Figs. 1B–1F). The microelectrodes were considered “active” if they detected at least 0.02 events per second. A network burst (NB) was defined as a major, network-wide, synchronization epoch (Figs. 1E–1F), during which at least 30% of active electrodes detected spiking activity in a 10 ms time window. The algorithm for network bursts identification considers a NB as finished when, in a 500 ms window, 80% of the activity remains below the detection threshold, considers NBs with an inter-burst interval of less than 500 ms as a single NB and requires a minimum NB duration of 100 ms. The onset and offset of each NB were defined as the points in time preceding and following the NB peak activity, respectively. More specifically, these were the points where the Gaussian-smoothed network-wide spike-time histogram (STH, i.e., estimated using 1 ms bins) reached zero. Basic statistics such as NB frequency, duration, and inter-burst intervals were derived from the onset and offset of the NBs if at least 20 NBs were detected during a recording session. The time course of the firing rate during each NB (i.e., STH) was further examined in terms of its slope at onset and of its spectral (dominant) frequency, during the offset NB trajectory following its peak, if present. In detail, the rising phase of the STH, prior to the NB activity peak, was best-fit by a double exponential function with equation $\mathrm{f} \left( \mathrm{t} \right) ={\mathrm{a}}_{0}{\mathrm{e}}^{ \left( {\mathrm{b}}_{0}-1 \right) \mathrm{t}}+{\mathrm{a}}_{1}{\mathrm{e}}^{ \left( {\mathrm{b}}_{1}-1 \right) \mathrm{t}}+\mathrm{d}$ using the non-linear least-square optimisation method. The smallest of the two exponents (b_0_ or b_1_) was then used to describe the rate of increase of network firing during the ignition phase of each NB. This value was obtained after averaging successive NB profiles and referred to each experimental specific condition and to each MEA.

Oscillatory frequency content in the offset phase of each NB was apparent in some, but not all MEAs (Pulizzi et al., 2016). This was revealed by pre-processing the data and individually aligning each NB prior to averaging, maximising similarity, as described previously. Frequency-domain analysis was then performed to extract the “dominant” frequency of the oscillation, by estimating the spectrogram (Oppenheim, Schafer & Buck, 1999) of each STH. In detail, the Fast Fourier Transform was employed to extract the time-varying spectrum of frequencies contained in the STH (Mattia, Ferraina & Del Giudice, 2010), averaging over all NBs. The “dominant” component of the power spectrum was defined as the Fourier frequency corresponding to the highest peak in the spectrum, which exceeded the median value of the spectrum by at least threefold.

All the parameters extracted from the data were monitored over 30 min time windows, in order to assess whether or not the stationarity of the recording was affected by the compound application. Indeed, the SP inhibitor compound could have short-term, persistent or delayed effects and care was taken to discriminate its time course. Furthermore, given the inherent variability of neuronal cultures, the relative changes in each parameter with respect to baseline, and not the absolute values, were chosen as the most important observables. Thus, each parameter was normalised to its respective value during the baseline recording conditions.

The effective connectivity was extracted from each experiment by means of conventional cross-correlation analysis of spike times (Knox, 1981). Briefly, for each spike detected at electrode A, the statistics of relative time delays of all the spikes detected at electrode B were computed. This analysis was repeated over all spikes detected at A and restricted to delays smaller than 150 ms. Then the overall distribution of delays was estimated by a histogram with 3 ms bins and its profile was subsequently referred to as the cross correlogram, which quantified the effective coupling between each pair of MEA microelectrodes. Microelectrodes detecting too few spikes (*n* < 150) were excluded from this analysis.

As the persistent effects of SP inhibition was of interest in this study, the cross-correlation analysis was limited to the last half hour of each recording session. For the baseline condition, the entire duration of the recordings was employed to increase statistical confidence. Each cross correlogram was normalised by the factor $1/\sqrt{{N}_{A}.{N}_{B}}$ , where *N*_*A*_ and *N*_*B*_ are the number of detected spikes for electrode *A* and *B* respectively. To represent the strength of the functional connection between each pair of microelectrodes, the peak value of the cross correlogram, C, was derived. Finally, the change in functional connectivity in response to the SP inhibitor treatment was expressed as a ratio ΔC = C_after_∕C_before_, where C_before_ is obtained from baseline conditions.

Intrinsic excitable cell membrane properties were finally studied by patch-clamp experiments. We specifically quantified the resting membrane potential, the average threshold and peak amplitude of the action potentials (AP), the apparent input membrane resistance, the membrane time constant, and the AP frequency *versus* stimulus intensity curve. In brief, the resting potential was defined as the median value of the recorded intracellular electrical potential, over 5 min while digitally excluding samples corresponding to spontaneous APs. The AP threshold and amplitude were computed from electrically evoked firing activity of the neurons, upon the injection of current-clamp square waveforms. These were defined as the maximum of the third derivative of the potential, and by the relative distance between the AP overshoot peak and threshold value, respectively. The membrane input resistance was calculated by a linear fit of the potential differences caused by hyperpolarising step currents of increasing absolute amplitudes. The membrane time constant was computed by fitting the recovery-to-baseline membrane potential trajectory, which followed a very brief hyperpolarising current pulse, with a double exponentials function. Finally, the AP frequency-current (FI) curve was constructed upon counting the number of evoked APs, elicited by depolarising current pulses of increasing amplitudes. Each FI curve was summarized by its slope, which was computed as the slope of the best-fit straight line over the non-zero values of the curve.

Statistical analysis was performed using MATLAB and it consisted of the Mann–Whitney *U* test to assess significant differences between treated and control groups.

## Results

A hallmark feature of the electrical activity of dissociated neuronal cultures is their tendency to spontaneously synchronise neuronal discharges, in the episodic occurrences of “bursts” of action potentials hereafter referred to as network-wide bursts (NB). Each NB lasted several hundreds of milliseconds (Kamioka et al., 1996) and recruited a large part of the network, as it was detected across many microelectrodes of the same MEA. During the onset of the NB, there was an exponential increase of the neuronal firing rate, as a dynamical reflection of the considerable structural recurrent excitation, which effectively represents a positive feedback loop in the system. The impact of the recruitment of inhibitory neurons became apparent at a later stage, together with the activation of several intrinsic and synaptic adaptation mechanisms, as the NB profile decayed and the network became ultimately silent, by an effective negative feedback loop in the system  (Weihberger et al., 2013). The main features of these NBs, such as their duration and occurrence frequency, are therefore directly related to the mutual interaction and balance between excitation and inhibition in the network. In turn, the interactions between the excitatory and inhibitory subpopulations are directly determined by their synaptic connectivity and by their short- and long-term plasticity.

In our experiments, we investigated the effects of serine protease inhibition on neuronal network activity *in vitro* as well as on single-cell excitability and intrinsic electrical properties. We repeated our experiments over *n* = 31 distinct neuronal cultures where the SP inhibitor was bath applied, and we compared the results from several electrophysiological observables in *n* = 29 more neuronal cultures, employed as the control condition.

**Figure 2:**
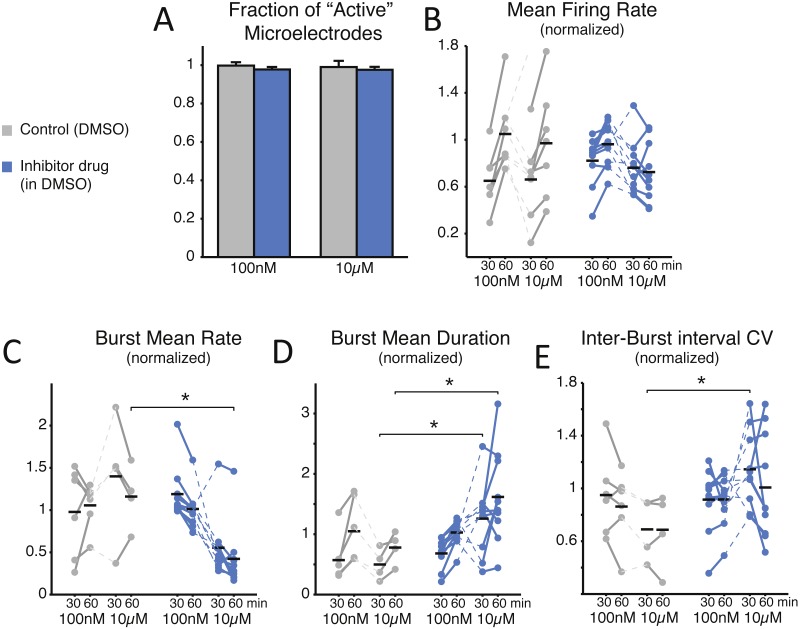
SP inhibition depresses network electrical activity, leading to a reduced NB occurrence rate, higher variability of inter-burst time intervals, and increased NB duration. The SP inhibition does not affect neural viability as the average number of *active* microelectrodes (A), normalised to baseline condition, is left unaltered (blue: treated MEAs, *n* = 10; grey: control MEAs, *n* = 7; error bars are the s.e.m.). The mean firing rate over the entire MEA is displayed in (B), normalised to and estimated during the first and last 30 min time intervals of bath-application of the SP inhibitor, showing no significant difference (black horizontal bars are the group averages). Instead, the inhibitor depresses the episodic occurrence of NBs, in cultures that display such a spontaneous activity pattern (10 treated and 6 controls at 100 nM, and 10 treated with 4 controls at 10 µM): the mean NB rate, normalised to baseline, decreases particularly at the high dose of the inhibitor (C). Correspondingly, the NB duration increases significantly (D) at the high dose. Finally, the coefficient of variation of the inter-burst time interval distribution (E), normalised to baseline, increases particularly at the high dose of the inhibitor.

Aiming at a preliminary indication of the potential toxicity of our chemical compound, we first examined the number of “active” microelectrodes, defined as those recording channels of each MEA that detected neuronal activity above the minimal rate of 0.02 events/s (Fig. 2A). We found no significant effect caused by the SP inhibitor and we observed that all MEAs had an absolute number of active electrodes exceeding 90% (99.8 ± 1.8% in control and 97.8 ± 1.1% in low-dose treated MEAs, *p* = 0.909; 99.0 ± 3.2% for control and 97.6 ± 1.5% in high-dose treated MEAs, *p* = 0.762). These findings indirectly suggest that the compound does not markedly harm the neurons or negatively interfere with intrinsic membrane properties underlying synaptic input integration and the generation of action potentials. Such an observation was later confirmed directly, by single-cell patch-clamp experiments (see below).

To gain an insight into the compound’s effect on the structural synaptic connectivity, some basic observables were chosen to quantify neuronal firing and network bursting from the recorded spike trains under each condition (Figs. 2B–2E). Despite the potency and strong selectivity of action, the SP inhibitor did not induce any appreciable effect when used at a low concentration (100 nM). In fact, when expressing all the observables as normalised to baseline conditions, in the final 30 min period the firing rate was 1.049 ± 0.124 in control, 0.962 ± 0.058 for the treated group (*p* = 0.0962); the NB rate was 1.055 ± 0.110 in control, 1.013 ± 0.074 in treated MEAs (*p* = 0.313); the NB duration was 1.049 ± 0.217 in control, 1.028 ± 0.068 in treated cultures (*p* = 0.958); and the coefficient of variation of the inter-burst intervals was 0.853 ± 0.113 in control, 0.916 ± 0.061 in treated cultures (*p* = 0.875). However, when used at a high final concentration (10 µM), the periodic character of the *in vitro* network bursting changed significantly (Figs. 2C–2E). This was the case, despite that neurons individually had similar firing rates (Fig. 2B; 0.661 ± 0.135 in controls and 0.762 ± 0.073 in treated cultures during the first 30 min after treatment, *p* = 0.601; 0.970 ± 0.177 in controls and 0.725 ± 0.081 in treated cultures in the second 30 min after treatment, *p* = 0.315). In fact, the occurrence frequency of the NBs decreased (Fig. 2C; 1.160 ± 0.187 in controls and 0.422 ± 0.120 in treated cultures, *p* = 0.014 < 0.05), while their duration increased (Fig. 2D; in the first 30 min, 0.498 ± 0.130 in controls and 1.259 ± 0.187 in treated cultures, *p* = 0.036 < 0.05; in the second 30 min, 0.778 ± 0.133 in controls and 1.619 ± 0.243 in treated cultures, *p* = 0.024 < 0.05), and their timing became more irregular, as indicated by an increase in the coefficient of variation of the inter-burst interval (Fig. 2E; 0.690 ± 0.119 in controls and 1.145 ± 0.090 in the treated group, *p* = 0.024 < 0.05).

We note that the control cultures displayed proportionally a qualitatively larger inter-individual variation in the firing rate and the NB frequency, compared to their baseline activity. The decreased NB frequency and increased irregularity of NB timings was significant (*p* < 0.05) for the second and first 30 min after drug administration, respectively.

**Figure 3:**
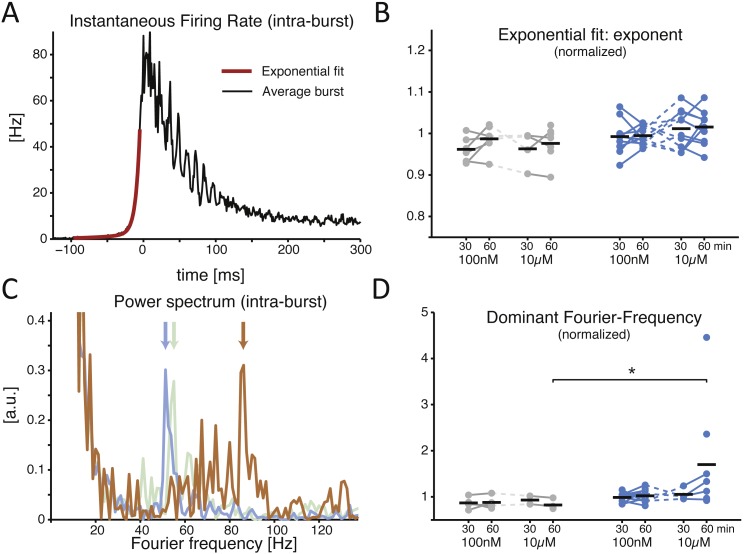
The SP inhibitor alters the features of the network burst at its offset, not onset. A representative example of the average instantaneous firing rate during a NB is displayed (A), estimated by a spike time histogram (STH). At onset the time course is best fit by an exponential function, while at offset it is often (not always) characterised by intra-burst oscillations. When the exponential onset increase in the firing rate is quantified, (B) by comparing the exponent of the best fit rising phase of the STH, normalised to baseline, the application of the inhibitor revealed no effect (black horizontal bars indicate the group averages). The power spectrum of the offset of the STH (C) quantifies an obvious transient oscillatory behaviour: coloured traces show the power spectrum density (PSD) for the baseline (grey), 100 nM (blue), and 10 µM (orange) for the same MEA, and the location by a coloured arrow of the *dominant frequency*. When the latter is normalised to baseline and compared across conditions, a significant increase is found at the high dose of the inhibitor (D).

When examined in its time course, the recruitment rate of the neuronal network during NB initiation followed an exponential growth (Fig. 3A) (Eytan & Marom, 2006). Quantified by the best fit of a double exponential function, the recruitment rate (Fig. 3A, red trace) of both control and treated cultures relative to baseline showed similar rise times (Fig. 3B; during the final 30 min period, 0.987 ± 0.012 in controls and 0.995 ± 0.007 in cultures with low dose, *p* = 0.962, and with high dose controls 0.976 ± 0.016 and 1.015 ± 0.013 for the treated group, *p* = 0.070). As soon as successive NBs, recorded in the same MEA, were aligned for maximal similarity of their temporal profile and averaged (see the Methods)  (Pulizzi et al., 2016), a prominent oscillation in instantaneous rate of neuronal discharge became apparent during the falling phase of the NBs (e.g., Fig. 3A) in several but not all cultures. This is apparent from the structure of the power spectrum (Fig. 3C) and it is thought to arise from the interplay between the excitatory and inhibitory neuronal populations as in the Pyramidal-InterNeuron Gamma-range brain rhythms emergence *in vivo* (PING) (Wang, 2010). When the “dominating” peaks (see the Methods) of the power spectrum were extracted (Fig. 3C), a significant increase in their Fourier frequency location could be observed, but only half an hour after the application of the inhibitor at the high dose (Fig. 3D; 0.824 ± 0.053 in controls and 1.701 ± 0.428 in the treated group, *p* = 0.028 < 0.05). As not all cultures revealed a clear-cut emergence of a dominant oscillation in the discharge rate of neurons during the NBs, cultures lacking oscillations in their corresponding baseline recordings were excluded from the analysis.

**Figure 4:**
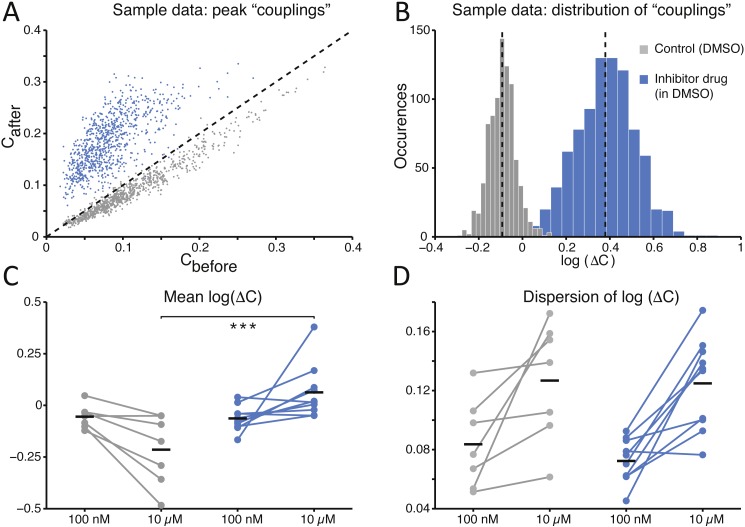
The average *functional coupling* across any microelectrode pair appears strengthened under SP inhibition. When compared to control conditions (grey), the strength of *functional coupling* increases (A) with the inhibitor at the high dose (blue). Each dot in the scatter plot corresponds to one pair of microelectrodes and displays the value of the peak of the spike cross-correlogram before and after the SP inhibition: dots above the unitary slope line imply an increase in strength with respect to baseline. The actual distribution of values, in the same representative example of panel (A), can be better displayed in logarithmic scale (base 10) while expressing the change (B) after the dose referred to the baseline: the black dotted vertical lines are the mean values of the distributions (control *versus* high dose) and are significantly different. Across all our experiments (C), 10 µM of the SP inhibitor significantly strengthened the functional couplings between microelectrodes compared to the control, while the latter tends to even decrease over time (black horizontal bars indicate the group averages). The standard deviation or *dispersion* (D) of the log_10_(ΔC) distributions increases over time, but in the same manner comparing the effect of the compound and the control.

Finally, to quantify the effects of SP inhibition on synaptic connectivity, the strength of functional connections (see the Methods) was estimated in both culture groups upon correlation analysis of the spike trains detected at distinct microelectrodes within the same MEAs, as in Eytan et al. (2004). As displayed for a representative experiment (Fig. 4A), functional connectivity was significantly altered by the SP inhibition when compared to the (physiological) spontaneous redistribution of the coupling strength over the same amount of time  (Mahmud et al., 2014). When quantified over the entire set of experiments, the change in coupling strength (ΔC) was referred to baseline conditions and averaged across all possible microelectrode pairs (Fig. 4B). A highly significant increase in ΔC was observed in its mean (Fig. 4C; −0.214 ± 0.064 in controls and 0.063 ± 0.041 in the treated group, *p* = 0.000103 < 0.001) although not in its dispersion (Fig. 4D; 0.127 ± 0.015 in controls and 0.125 ± 0.010 in treated cultures, *p* = 0.669), suggesting that stronger effective coupling appeared over the course of the time interval following the application of the inhibitor.

**Figure 5:**
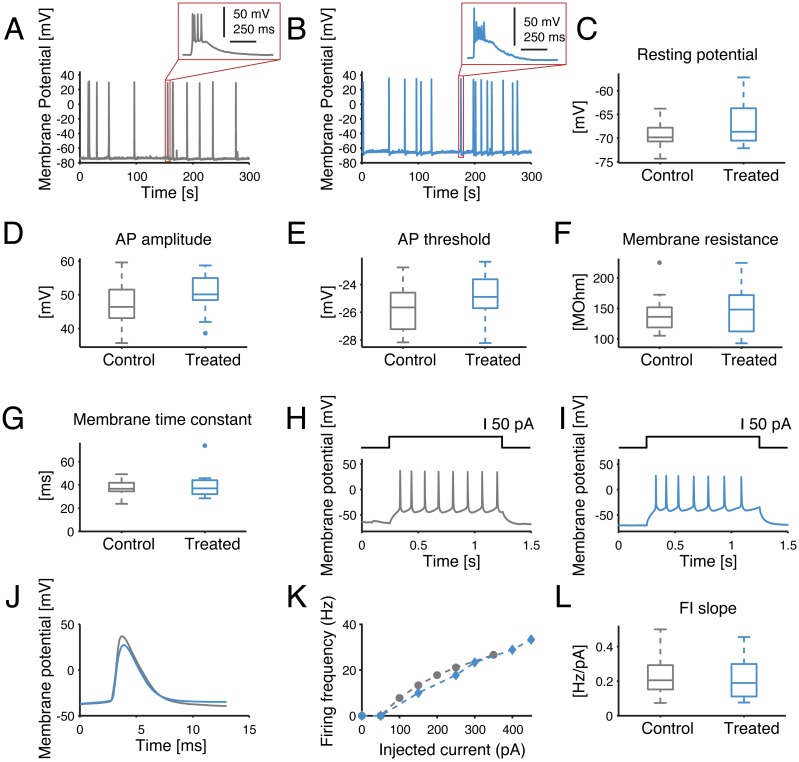
Single-cell excitability and intrinsic membrane electrical properties are not affected by the SP inhibitor. (A–B) Representative intracellular recordings, performed under current-clamp, are displayed for control (grey) and SP inhibition (10 µM, blue). Spontaneous APs occur in bursts for both conditions, as seen in the insets. (C–G) No significant difference was found for the resting potential, average AP amplitude (i.e., from threshold to peak value), average AP threshold, membrane resistance or membrane time constant. (H–I) Representative example of evoked current-clamp AP firing: cells respond in a similar fashion to an injected 50 pA step current. (J) The comparison between the first evoked AP reveals no significant effect of the SP inhibitor on the AP waveform. (K) Representative example of AP frequency *versus* current intensity (F–I) curves, for both control conditions and SP inhibition. (L) The slope of the F–I curves of the control *versus* treated cells is not significantly different.

To further investigate whether the observed network-level effects could be attributed to changes in single-cell properties, intracellular patch-clamp experiments were performed in the whole-cell configuration. In fact, a reduction in the single-cell excitability would account for the lower occurrence frequency of NBs and their delayed termination, as individual neurons would be less likely to be recruited in each NB. However, none of the membrane passive or active cellular properties were affected by the SP inhibition, half an hour after bath application of the compound (Fig. 5). The resting potential, membrane resistance, membrane time constant, AP amplitude, AP threshold or FI slope were in fact statistically indistinguishable for cultures treated with the control solution or with 10 µM of the inhibitor (*p* > 0.05).

**Figure 6:**
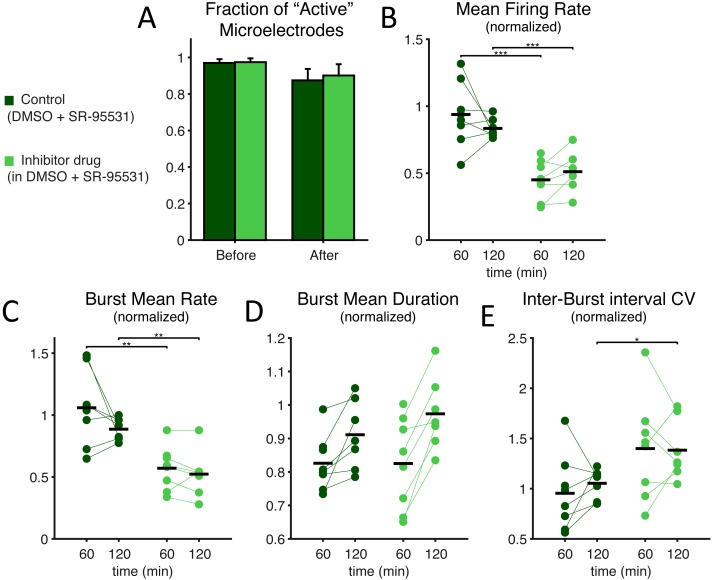
Under GABA_A_ receptor blockade too, SP inhibition depresses network electrical activity, leading to a reduced NB occurrence rate, higher variability of inter-burst time intervals, increased NB duration, but also to an overall decreased mean firing rate. Under concurrent bath application of a selective antagonist of GABA_A_ synaptic receptors (SR-95531), the effects of SP inhibition at the highest dose were very similar to what was reported in Fig. 2, in terms of *active* microelectrodes (A) (light green: treated MEAs, *n* = 8; dark green: control MEAs, *n* = 8; error bars are the s.e.m.), NB rate decrease, NB mean duration, and inter-burst time interval variability. The mean firing rate over the entire MEA, displayed in (B), and normalised as in Fig. 2, shows a significant decrease when compared to control (black horizontal bars are the group averages).

**Figure 7:**
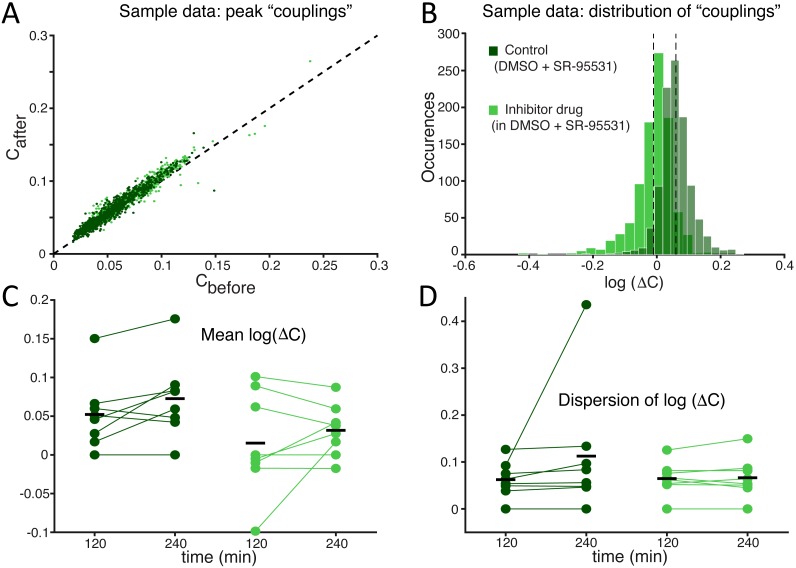
Under GABA_A_ receptor blockade too, the competing effects of SP inhibition and of a mean firing rate reduction compensate each other, revealing no significant differences in the average *functional coupling* under SP inhibition. As we repeated the experiments of Fig. 4 in the presence of a selective antagonist of GABA_A_ synaptic receptors (SR-95531), the effects of SP inhibition on neuronal functional couplings (A–B) were masked by the decreased mean firing rate (Fig. 6). Indeed, cross-correlation analysis is sensitive to changes in the neuronal firing frequency, so that a drop in the mean firing rate corresponds to decreased functional coupling. As we observed no change in the mean values and dispersion (C–D) of the cross-correlation peaks, the positive effect of SP inhibition must have persisted under GABA_A_ receptors blockade and appeared masked by the negative firing rate dependent effect.

Finally, we explored SP inhibition under pharmacological isolation of the excitatory neuronal subpopulation, upon bath application of a selective antagonist of GABA_A_ synaptic receptors (Figs. 6–7). Under such conditions, we found that SP inhibition still depressed network electrical activity while the fraction of active electrodes did not significantly differ in the presence or absence of the SP inhibitor (Fig. 6A; from 0.970 ± 0.059% to 0.875 ± 0.176% in control and from 0.975 ± 0.059% to 0.901 ± 0.175% in high-dose treated MEAs, *p* = 0.96 and 0.64 respectively). More in the details, the occurrence frequency of the NBs decreased (Fig. 6C; during the first hour, 1.059 ± 0.300 in controls and 0.571 ± 0.174 in treated cultures, *p* = 0.0019, and during the second hour, 0.775 ± 0.323 in controls and 0.523 ± 0.186 in treated cultures, *p* = 0.022), and their timing became more irregular, as indicated by an increase in the coefficient of variation of the inter-burst interval (Fig. 6E; 1.054 ± 0.146 in controls and 1.383 ± 0.299 in treated cultures during the second hour, *p* = 0.011). However, the mean-firing rate also decreased (Fig. 6B; during the first hour, 0.938 ± 0.239 in controls and 0.450 ± 0.145 in treated cultures, *p* = 0. 00062, and during the second hour, 0.730 ± 0.302 in controls and 0.447 ± 0.226 in treated cultures, *p* = 0. 0071). A decrease in the mean firing rate has been reported to negatively affect the cross-correlation between spike trains (De La Rocha et al., 2007). When repeating the experiments of Fig. 4 in the presence of the GABA_A_ receptor blocker, SP inhibitor boosting of effective connectivity should be masked by the effect of the decreased firing rate. This was indeed the case (Fig. 7) as we observed no significant effects of the SP inhibitor in our cross-correlation analysis.

## Discussion

The ECM is a key player in the regulation of intrinsic excitability and structural synaptic connectivity, although the involvement of its different molecular constituents is largely unknown. Proteolytic regulation of ion channels is associated with the ECM (Wójtowicz, Brzdak & Mozrzymas, 2015) and the mechanisms underlying action potential generation (e.g., voltage-gated sodium channels) are also implicated in adhesion, migration, path finding, and transcription (Bourgognon et al., 2013).

We investigated the indirect effect of a serine protease inhibitor on network- as well as single cell-levels electrophysiology, with a high binding affinity for two important regulators of the ECM: uPA and neuropsin. We observed significant alterations in the collective emergent activity *in vitro* within just 1 h following the application of the SP inhibitor, consistent with the rapid *in vivo* action of ECM proteases (i.e., over minutes to hours (Wójtowicz, Brzdak & Mozrzymas, 2015)). As we found the SP inhibitor left single-cell excitability and intrinsic properties unaltered, the effects observed on network bursting may be linked with increased confidence to glutamatergic and GABAergic synaptic transmission, as a downstream point of regulation by ECM proteases.

The increase of the average NB duration, the decrease in the NB occurrence, the increase in inter-burst variability, and the increase in the oscillation frequency within NBs indicate a shift of the balance in excitation and inhibition towards the latter. A weakened excitation (or a relatively higher inhibition) would allow the recurrent excitation to ignite a NB less frequently and more randomly over time and, once started, to let it carry on for longer while delaying its termination. This has been illustrated in an early *in silico* study by Giugliano and collaborators (Giugliano et al., 2004) and also *in vitro* by Gross and collaborators, who boosted inhibition through administration of GABA and decreased NB duration (Gross, Rhoades & Jordan, 1992). A shift towards inhibition is also predicted by a mean-field model of the network activity, leading to faster intra-burst oscillations (Pulizzi et al., 2016). There, an increase of the efficacy of inhibitory connections or their numbers increases the intra-burst frequency oscillation.

To further directly investigate how the observed change in activity relates to the glutamatergic and GABAergic synapses, experiments were repeated in the presence of a selective pharmacological antagonist of GABA_A_ receptors. As the contribution of GABAergic neurons to the overall synaptic inputs to a neuron was silenced, effects of the SP inhibitor on NB that still persisted in the presence of the blocker could be attributed to the interaction of the SP inhibitor with excitatory neurons. Vice versa, NB effects that did not persist in the presence of the blocker could be attributed to synaptic inputs from GABAergic neurons. Both the reduction of NB rate and the increase in the inter-burst interval variability persisted under GABA_A_ receptors blockade, indicating that the drive of the glutamatergic population was diminished by SP inhibition. The effect of the SP inhibitor on the NB duration instead disappeared under GABA_A_ receptors blockade, indicating that SP inhibition led to a decreased GABAergic synaptic drive as well.

Two important network elements where the basis of this change in activity could lie are the synaptic connections and the intrinsic neuronal cell properties. However, should the SP inhibitor affect single-neuron properties, changes at the level of intrinsic membrane excitability would be expected as measured by the patch-clamp technique. This was not the case as no significant effect of SP inhibition could be found at the level of cellular properties. As for the synaptic connections, the significant increase in the action potential firing synchrony through cross-correlation analysis—despite being only an indirect measure of effective and not anatomical coupling—indicates synaptic connections are strengthening overall. The increased frequency of intra-burst oscillations in the instantaneous firing rate of the network is consistent with an increase in the glutamatergic synaptic efficacy as well as with an increase in the GABAergic synaptic efficacy (Pulizzi et al., 2016), in line with the functional connectivity results. GABAergic neurons, when recruited by excitatory neurons, operate with a dampening effect on the network’s explosive behaviour during an NB.

Taken together, the findings on network bursting dynamics both with and without GABAergic blockade, and including the analysis of functional connectivity, indicate that while on average connections are strengthening, the activity of both populations is decreasing.

Interestingly, evidence from the literature indicates a shift of the E/I balance towards excitation in response to a decrease in certain SP levels. Complete deficiency of neuropsin has been shown to shift the E/I balance *in vivo* towards excitation, in the hippocampus of mice treated with kainic acid, thus leading to a more severe progression of the status epilepticus than in control (Kawata et al., 2017). Similarly, in an animal model of epilepsy, a pathological condition associated with strong recurrent excitation, it was recently found that the frequency of seizures is higher in epileptic rats that show a more pronounced decrease in active uPA and neuropsin (Missault et al., 2017). While our results do not unanimously point to a shift of the E/I balance in either direction, the SP inhibition certainly had a profound effect on the dynamics of the network.

Furthermore, when tested at lower concentrations such as 100 nM and 1 µM (not shown), the compound demonstrated no noticeable effects, despite being several times above the IC_50_ for uPA, neuropsin (KLK8) and KLK4 (i.e., 21, 15, and 44 nM, respectively (Missault et al., 2017)). It is therefore likely that at the 10 µM concentration we used, the compound is (partially) inhibiting additional serine proteases alongside uPA and neuropsin. Indeed, in human tissue, the inhibitor displays affinity in the nanomolar range for cathepsin G, tryptase, and matriptase, and in the micromolar range for KLK1, tPA, thrombin, Factor Xa, HNE and AChE. Additionally, uPA expression levels were shown to be low in murine CNS under normal conditions (Sappino et al., 1993), only being upregulated during pathological conditions (Lahtinen, Lukasiuk & Pitkänen, 2006; Gorter et al., 2007).

An initial limit of our study was the use of a cell culturing protocol based on heat-inactivated horse serum, complementing the culture medium. Since the serum is chemically not defined, it is conceivable that exogenous SP, if present in the serum, may have been sequestering the inhibitor, explaining the need for higher concentrations than suggested by the IC_50_. To this end, experiments were repeated under serum-free conditions (File S1). We found that while at 10 µM there was a clear effect on the network’s electrophysiology, 1 µM still had no effect on any of the quantified parameters (File S1). This suggests that in cultures with serum, the SP inhibitor is not being significantly sequestered by unknown serum proteins. It is also conceivable that the serum might contain SP inhibitors, so that serum-free cultures should then show a reduced effect when the SP inhibitor was applied. We found that the effects on the network’s electrophysiology was even stronger, as not only NB rate was depressed (File S1—Fig. S1C) but also the overall mean firing rate decreased in the presence of the inhibitor (File S1—Fig. S1B).

Despite our best attempts, neuronal culture health and quality were seriously affected by removing serum. The likelihood of cultures surviving beyond 3 weeks *in vitro* was low and the plating cell density had to be reduced from 6’500 to 2’000 cells/mm^2^ to improve survival. Additionally, serum-free cultures often displayed *superbursts*, constituted by single NBs lasting several seconds, much longer than regular NBs. This kind of activity has been described in developing cultures, around DIV7 to 14 (Wagenaar, Nadasdy & Potter, 2006), thus indicating that our serum-free cultures have a delayed development. For all these reasons, any more in depth quantitative comparisons between the specific results obtained in serum-containing and in serum-free cultures should be regarded with care.

## Conclusion

We have investigated the effect of serine protease inhibition on the electrophysiological activity of dissociated cortical networks through the use of intracellular patch-clamp recordings and of MEA technology. The results demonstrate that such a pharmacological inhibition alters the activity dynamics of the culture, specifically the network bursting and the functional connectivity. No changes were observed in single-cell excitability when passive and active membrane electrical properties were studied. We conclude that interfering with the ECM by the inhibitor changed the effective connectivity of the network, resulting in an altered activity level of the excitatory and inhibitory populations. Serine proteases have a known important role in the modulation of the ECM, suggesting that the changes in network dynamics we observed are related to the target pathways in the ECM of serine proteases. Further studies are required to identify the exact targets and pathways through which this inhibitor changes the pattern of activity in the network. A more detailed identification of all the molecular players in the ECM and of their roles in regulating neuronal connectivity would further contribute to unravel the mechanisms of brain wiring.

##  Supplemental Information

10.7717/peerj.6796/supp-1 File S1 Supplementary material detailing the serum-free culturing protocol and the additional electrophysiological analysis repeated under those conditions

## Additional Information and Declarations

### Data Availability

The following information was supplied regarding data availability: Relevant data sets and analysis scripts, including an index of the deposited data, are available at Giugliano, Michele; Van De Vijver, Sebastiaan (2019): The Effect of Pharmacological Inhibition of Serine Proteases on Neuronal Networks *in vitro*. figshare. Fileset. https://doi.org/10.6084/m9.figshare.5873685.v2.
