# Supplementary material for: The effect of pharmacological inhibition of Serine Proteases on neuronal networks in vitro
Source: PeerJ. 2019 Apr 23;7:e6796. doi: 10.7717/peerj.6796 (PMC6485206; doi:10.7717/peerj.6796)
Supplement: File S1 [file peerj-07-6796-s001.pdf]

Supplementary File 1

The Effect of Pharmacological Inhibition of Serine Proteases on Neuronal Networks in vitro

Sebastiaan Van De Vijver, Stephan Missault, Jeroen Van Soom, Pieter Van Der Veken, Koen Augustyns, Jurgen Joossens, Stefanie Dedeurwaerdere, Michele Giugliano*

*michele.giugliano@uantwerpen.be

Supplemental Methods

Serum-free neuronal cultures. The data referring to 10 μM SP inhibitor originated from 35 pups over 6 cell culturing sessions, while the data referring to 1 μM originated from 12 pups over 2 culturing sessions. All the cultures were produced, maintained, and handled as described in the main text, with the exception of the following elements. The plating density was reduced to 2'000 cells/mm^2 to improve the rate of cell survival, over at least a period of 3 weeks after plating. Half-volume medium replacements were carried out every two days as for the normal cell culturing protocol. However, fresh medium without serum was employed in this case, aiming at a slow reduction of the serum content in the medium. Cells cultured without serum were exposed to neurobasal medium, supplemented with 10% B-27, 50 μg/mL gentamicin and 0.5 mM L-glutamine. To further increase survival rate, 5% heat-inactivated horse serum was added to the culture medium but solely on the day of cell plating. Through each medium change, the concentration of serum in the medium was gradually reduced over a period of 18 days.

Pharmacology and experimental protocol. Serum-free MEAs were treated in a similar fashion as the serum-positive cultures, except that they were exposed to a single concentration of the inhibitor (1 μM or 10 μM), and that recordings continued for 2h after compound addition.

Extracellular electrophysiological recordings. The electrophysiological activity of serum-free MEAs was recorded at DIV20-22, using the same procedure and equipment described in the main text.

Supplementary Figures

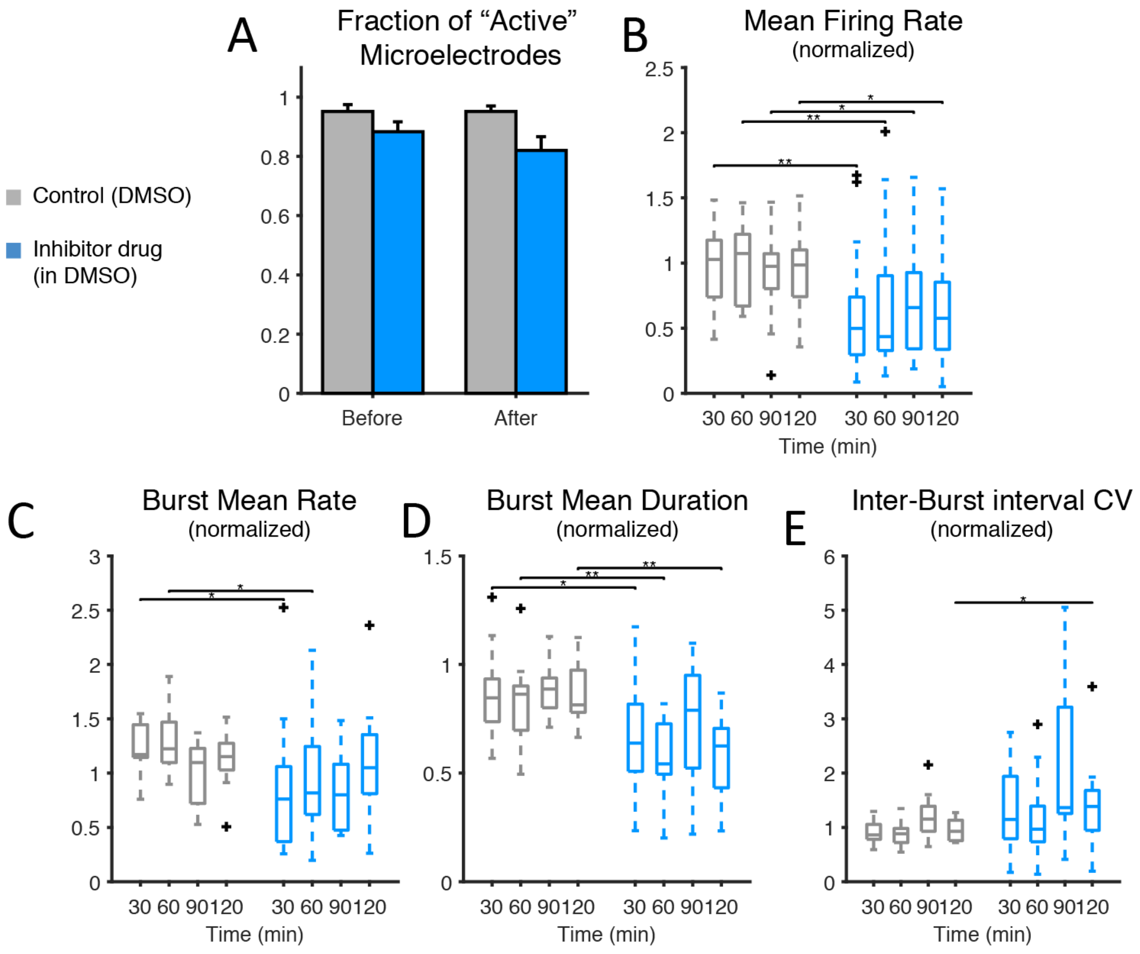

Supplementary Figure S1. SP inhibition significantly alters the network spontaneous electrical activity, in serum-free culturing conditions. To rule out that the undefined composition of the culture medium could significantly bias our results, we repeated the analysis described in Fig. 1 in serum-free conditions. Once again SP inhibition depressed NB occurrence and increased their variability. The compound does not affect neural viability as the average number of *active* microelectrodes (**A**), normalised to baseline condition, is left unaltered by the compound (blue: treated MEAs, n = 8; grey: control MEAs, n = 8; error bars are the s.e.m.). The mean firing rate over the entire MEA is displayed in (**B**), normalised to and estimated during four consecutive 30 min time intervals of bath-application of the SP inhibitor, shows a significant difference (black horizontal bars are the group averages). Moreover, the inhibitor alters the episodic occurrence of NBs, in cultures that display such a spontaneous activity

pattern (8 treated and 8 control): the mean NB rate, normalised to baseline, decreases (**C**). The NB duration increases significantly (**D**). Finally, the coefficient of variation of the inter-burst time interval distribution (**E**), normalised to baseline, increases.

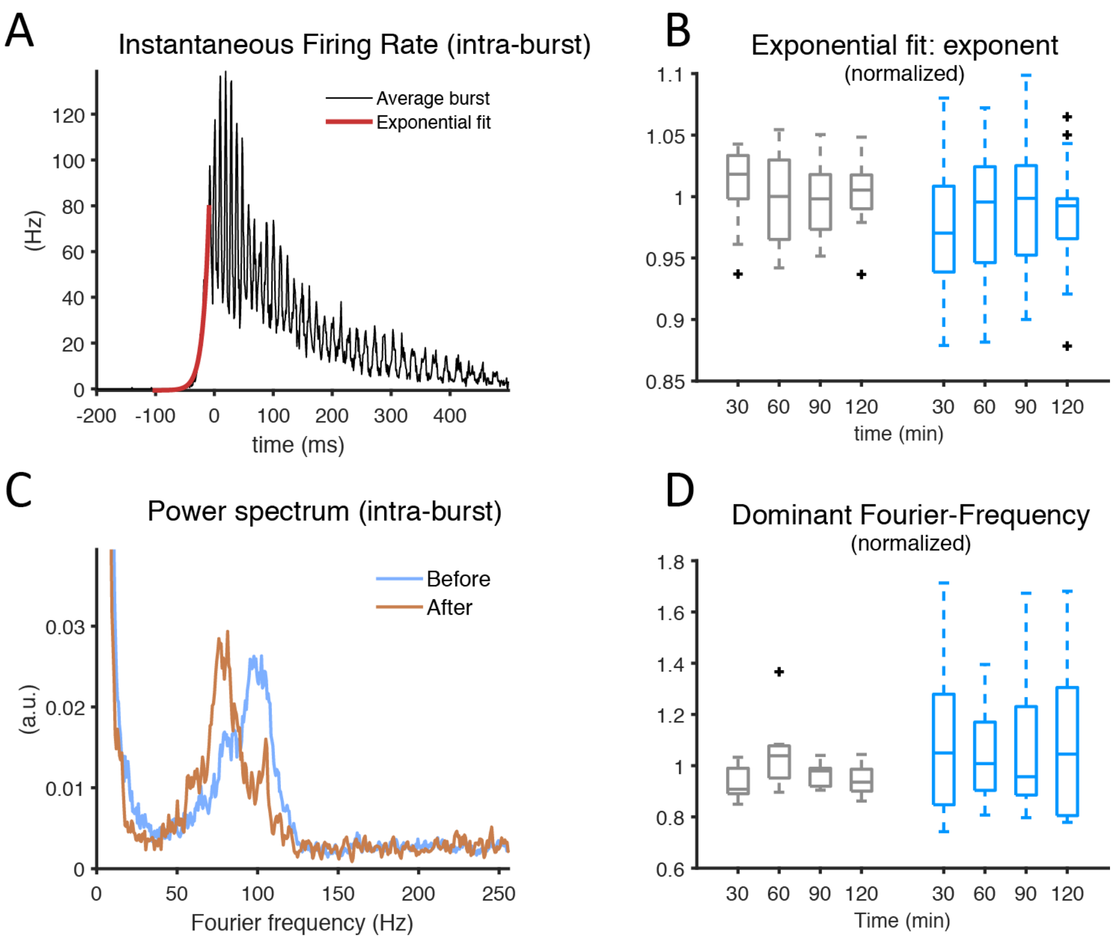

Figure S2. 10 µM of the SP inhibitor does not alter features of the network burst in serum-free cultures. A representative example of the average instantaneous firing rate during an NB is displayed (**A**), estimated by a spike time histogram (STH). At onset the time course is best fit by an exponential function, while at offset it is often (not always) characterised by intra-burst oscillations. When the onset is quantified, (**B**) by comparing the exponent of the best fit rising phase of the STH, normalised to baseline, the application of the inhibitor had no effect (black horizontal bars indicate the group averages). The power spectrum of the offset of the STH (**C**) quantifies an obvious transient oscillatory behaviour: coloured traces show the power spectrum density (PSD) for the baseline (grey) and 10 µM (blue) for the same MEA, and the

location by a coloured arrow of the *dominant frequency*. No significant change is found when normalising to baseline and comparing across conditions (**D**).

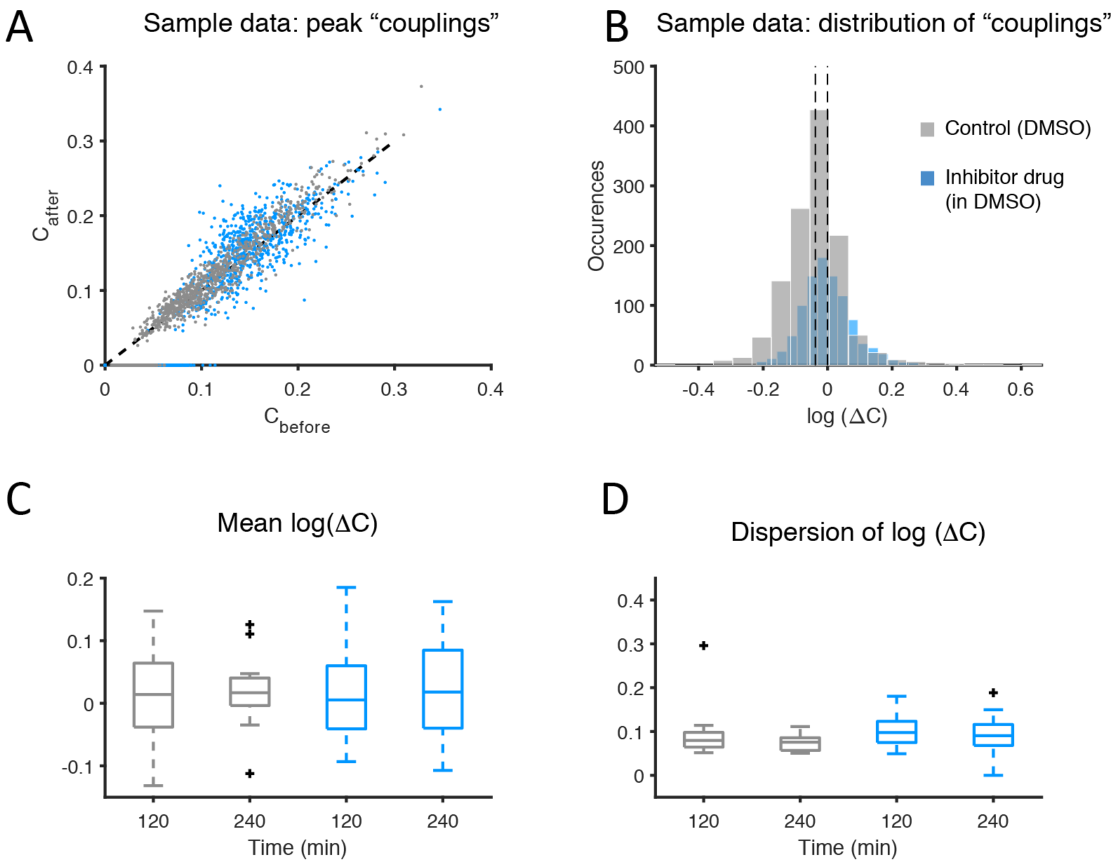

Figure S3. The average functional coupling across any microelectrode pair appears not to change under 10 μM SP inhibition of serum-free cultures. When compared to control conditions (grey), the strength of functional coupling as measured by cross correlation analysis does not change (**A**). Each dot in the scatter plot corresponds to one of the possible microelectrode pairs and displays the value of the peak of the spike cross-correlogram: dots above the unitary slope line imply an increase in strength with respect to baseline. The actual distribution of values, in the same representative example of panel (**A**), can be better displayed in logarithmic scale (base 10) while expressing the change (**B**) after the dose referred to the baseline ($\Delta C = C_{after}/C_{before}$): the dotted vertical lines are the mean values of the distributions (control versus high dose). Across all our experiments (**C**), 10 μM of the SP inhibitor did not significantly alter the functional couplings between microelectrodes compared to the control.

The standard deviation or dispersion (**D**) of the log10 (ΔC) distributions increases over time, but in the same manner comparing the effect of the compound and the control.
